# Telehealth Initiative to Enhance Primary Care Access in Brazil (UBS+Digital Project): Multicenter Prospective Study

**DOI:** 10.2196/68434

**Published:** 2025-04-29

**Authors:** Celina de Almeida Lamas, Patrícia Gabriela Santana Alves, Luciano Nader de Araújo, Ana Beatriz de Souza Paes, Ana Claudia Cielo, Luciana Maciel de Almeida Lopes, André Longo Araújo de Melo, Thais Yokoyama, Clarice Pagani Savastano, Paula Gobi Scudeller, Carlos Carvalho, Digital Primary Health Unit HCFMUSP Study Group

**Affiliations:** 1 Saude Digital Nucleo de Inovacao Tecnologica (InovaHC) Hospital das Clínicas, Faculdade de Medicina, Universidade de Sao Paulo (HCFMUSP) Sao Paulo Brazil; 2 Divisao de Pneumologia Instituto do Coracao (InCor) Hospital das Clínicas, Faculdade de Medicina, Universidade de Sao Paulo (HCFMUSP) Sao Paulo Brazil; 3 Brazilian Support Agency for Management of the Unified Health System (AgSUS) Brasília Brazil

**Keywords:** telehealth, public health service, innovation, primary health care, digital health

## Abstract

**Background:**

Brazil faces significant inequities in health care access, particularly in remote communities. The Brazilian Unified Health System is struggling to deliver adequate health care to its vast population. Telehealth, regulated in Brazil starting in 2022, emerged as a solution to improve access and quality of care. Thus, the *Hospital das Clínicas da Faculdade de Medicina da Universidade de São Paulo*, in partnership with the *Agência Brasileira de Apoio à Gestão do Sistema Único de Saúde*, created the *Unidade Básica de Saúde* (UBS)+Digital project, which aimed to mitigate the lack of medical care in remote areas of Brazil by providing teleconsultation in primary health units (PHUs) across the country. Through teletraining and digital health strategies, the initiative enabled health care professionals to provide remote assistance, improving access to medical care.

**Objective:**

To describe the implementation and results of the UBS+Digital project, a telehealth initiative focused on training health care professionals, providing teleconsultations, and monitoring key performance indicators among PHUs in Brazil.

**Methods:**

The study examined 15 Brazilian PHUs using a multicenter, prospective design. Data were collected through anonymous surveys of patients and physicians, which were recorded in the REDCap (Research Electronic Data Capture) database. PHUs were selected based on criteria such as the absence of an on-site physician and existing technological infrastructure. Synchronous and asynchronous training was provided, focusing on digital health and teleconsultation skills. In loco training included workshops and community events to share experiences and foster local engagement. A community of practice facilitated ongoing knowledge exchange. Teleconsultations followed the person-centered clinical method and Calgary-Cambridge methodology. Key performance indicators were monitored by a dashboard to guide continuous improvement. The transition of operations was managed based on physician availability and project duration. Microcosting analysis assessed the project’s economic impact using Brazilian guidelines, with statistical analysis performed using Jamovi software.

**Results:**

From March to November 2023, the project conducted 6312 telehealth sessions. A total of 342 professionals were trained, including participants from all three training modalities that were implemented. The Net Promoter Score for teleconsultations was 97, indicating excellent service quality. Of the teleconsultations, 65.3% (4009/6140) were prescheduled, and 34.7% (2130/6140) were on demand, depending on the family health team organization. Teleconsultations resolved 85% (5219/6140) of cases, with 15% (921/6140) requiring in-person referrals or emergency care. The average absenteeism rate was 15% (1083/7223), and consultation durations were between 15 and 20 minutes, suggesting potential adjustments in scheduling.

**Conclusions:**

The results highlight the effectiveness of telehealth programs in primary care settings with limited medical professionals. The UBS+Digital project demonstrated that telehealth can enhance health care access, presenting a pioneering model within the Brazilian Unified Health System for digital primary care.

## Introduction

Universal and free access to health care is constitutionally guaranteed by Brazil’s Federal Constitution of 1988 and its *Sistema Único de Saúde* (SUS; English: Unified Health System), established in 1990. Over its 3-decade history, the SUS has demonstrated significant advancements in promoting the Brazilian population’s right to health care, attending to over 190 million people annually at no cost and striving for higher quality service [1].

Health care services within SUS are organized according to the complexity needed to meet population demands: primary health care (PHC), medium complexity, and high complexity services. In this regard, the primary health unit (PHU; Portuguese: *Unidade Básica de Saúde* [UBS]) is a fundamental structure within the Brazilian health care system, designed to provide PHC and accessible, high-quality health services to the population. The *Estratégia Saúde da Família* (ESF; English: Family Health Strategy) team is a central component of this structure, emphasizing health promotion and disease prevention, treatment, and management through a community-based approach. The ESF is composed of a multidisciplinary team of health care professionals. This team, which is the minimum required, consists of a physician, a nurse, a nursing technician, and at least a community health agent [2,3].

The National Primary Care Policy determines that each ESF team should oversee no more than 4000 individuals, with an ideal average of 3000. This allocation should adhere to equity standards, as the number of individuals per team reflects the vulnerability levels of families in the region [4]. The PHC coverage in Brazil was reported at 78.56% as of September 2023, with more than 50,000 accredited ESF teams [5]. Considering that approximately 71.5% of Brazilians lack private health insurance, the SUS remains fundamental in medical treatments, health assistance, and comprehensive health care services [6]. In addition, PHC serves as the primary gateway for patients, addressing more than 85% of the health needs expressed by the population [7]. Due to Brazilian geography, economic disparities, and diverse population densities, the health care system faces the challenge of offering higher-quality health services [7]. Thus, telehealth initiatives have emerged to enhance health care accessibility by facilitating rapid diagnosis and early disease detection [8].

Telehealth, defined as the use of information and communication technologies (ICTs) to deliver health care services remotely, has been recognized as a crucial tool to enhance health care accessibility and quality [9]. In Brazil, telehealth was formally regulated in 2022, establishing guidelines for its practice (Brazilian Law, Nº 14.510, December 27, 2022). Since its regulation, telehealth has been increasingly implemented across various health care domains, particularly in PHC settings, where it is used for remote consultations, monitoring of chronic conditions, and facilitating specialist referrals [10]. Several studies highlight the potential of telehealth to bridge health care gaps, especially in underserved regions, by providing timely access to medical professionals [10]. A study conducted in Australia [11] examined health care professionals’ experiences with the rapid upscaling of telehealth during the pandemic. The study found that telephone consultations were the predominant method of telehealth delivery, while video consultations were less frequently used. Although telehealth improved access to care for vulnerable populations and those in rural areas, it posed challenges for patients with language barriers and limited digital literacy. Despite these limitations, most participants supported the continuation of telehealth with appropriate funding and improvements in care models.

In this context of telehealth expansion in Brazil, the *Hospital das Clínicas da Faculdade de Medicina da Universidade de São Paulo* (HCFMUSP) has established itself as a leading reference in the implementation of digital health services. The HCFMUSP is the top public hospital in Brazil and is considered a reference hospital in Latin America [1]. The HCFMUSP plays a significant role within the SUS, which serves over 200 million people and is one of the largest public health systems in the world. To expand health care coverage for the Brazilian population and reduce disparities in medical care, the HCFMUSP developed the Digital Health Program, which has become a leading reference for telehealth services in Brazil. Although teleconsultation was only regulated in Brazil after the COVID-19 pandemic, the HCFMUSP had already been conducting studies in digital health, which facilitated the swift integration and provision of teleconsultations as soon as this modality was officially authorized. Additionally, other telehealth projects have been developed by the HCFMUSP Digital Health Program based on its expertise, such as the tele–intensive care unit (ICU) and the smart ICU [12]. From a broader perspective, the HCFMUSP Digital Health Program projects have been based on 3 pillars: training health care professionals, providing tele-interconsultation (consultations conducted by 2 or more professionals to discuss clinical cases) or teleconsultation (consultations performed between physicians and patients), and managing key performance indicators (KPIs).

The digital health initiatives of the HCFMUSP in PHC began in 2021, driven by the need to address persistent challenges in this health care sector. A collaborative initiative between the HCFMUSP and the UK Government’s Better Health Program developed a Digital PHC proof of concept. This initiative focused on implementing telemedicine service in Santarem, Pará, Brazil, due to the lack of health care services in remote areas. This included teletraining medical and multiprofessional teams to independently operate in telemedicine service, adapting to the specific challenges and realities of the Brazilian context [8].

Between September and December 2021, as part of the Digital PHC proof of concept, we conducted over 220 teleconsultations, providing medical care to 111 patients. The results demonstrated high patient satisfaction, with approximately 94.6% (105/111) of participants expressing satisfaction with the service, and 76.6% (85/111) reporting that their medical needs were fully met [8]. Based on the Digital PHC proof-of-concept results, we expanded the project to include 15 additional PHUs. This expansion was carried out in partnership with the *Agência Brasileira de Apoio à Gestão do Sistema Único de Saúde* (AgSUS; an entity created to provide operational support for the implementation of public health policies formulated by the Brazilian Ministry of Health) and the HCFMUSP*.* The initiative was named UBS+Digital (English: PHU+Digital) and focused on assisting different regions across Brazil. Therefore, the UBS+Digital project aimed to provide medical care through teleconsultations for patients seeking assistance at PHUs but who were not attended to due to the absence of an on-site physician. Additionally, the project sought to demonstrate that patients who received care were satisfied with the service and that its implementation was feasible and accessible across different regions of Brazil. Thus, the objective of this study was to describe the implementation and results of the UBS+Digital project, a telehealth initiative focused on teletraining of health care professionals, providing teleconsultations, and monitoring KPIs among PHUs in Brazil.

## Methods

### Study Design

This study, conducted between March and November 2023, represents a multicenter, prospective study that describes the implementation of a telehealth program at 15 Brazilian PHUs.

### Ethical Considerations

The Brazilian National Health Council (resolutions 466/12 [13] and 510/16 [14]) establishes that an ethics committee review is not required for studies whose sole objective is the evaluation of implementation processes or service improvement. Therefore, according to the Ethics Committee of *Hospital das Clínicas, Faculdade de Medicina*, *Universidade de São Paulo* (*Comissão de Ética para Análise de Projetos de Pesquisa*), this project did not require ethical analysis by the national research ethics committee system (*Comitês de Ética em Pesquisa*). In this study, we conducted an anonymous survey with physicians to gather technical information about the provided service, ensuring that no clinical or sensitive patient data were collected. Data collection was conducted using the REDCap (Research Electronic Data Capture) database, and all information was anonymously recorded. All data collected and analyzed in this study were fully anonymized prior to use. No personally identifiable information was retained or included in any part of the dataset. The research team implemented strict data management procedures to ensure participant confidentiality throughout the study, in compliance with ethical and legal standards.

### Human Resources Structure

Family and community medicine physicians were scheduled for 18 hours per week. Each physician was responsible for attending one PHU, ensuring continuity of care. The physicians’ weekly schedule included 1 hour for PHU team meetings, 1 hour for educational upgrading, and 16 hours for patient care (including teleconsultations, patient reception, prescribing and sending referral requests, and reviewing guidance with the patient).

The project team also included project managers, project coordinators, project analysts, research coordinators, data analysts, and information technology technicians, all of whom dedicated 40 hours per week to the project. In addition, a medical project coordinator and a nurse teletraining analyst dedicated 20 hours per week to the project. All professionals underwent a structured onboarding program developed by the Human Resources of Digital Health at the HCFMUSP and completed a 20-hour “Digital Health at Primary Healthcare” training program (the course description is given in the “Training Program” section.

The selected project manager and project coordinators should have experience in PHC, both of whom were responsible for the overall management of the project. Their duties included financial planning, monitoring, and improving collected KPIs and serving as the interface with stakeholders at the PHUs. The medical and project coordinators were responsible for selecting the operational team for the UBS+Digital project. Two key criteria guided the selection process: expertise in PHC and soft skills, such as decision-making, problem-solving, and leadership. The research team, including the research coordinator and the data analyst, was responsible for preparing reports, writing scientific materials, and managing the collected data. The teletraining team, composed of nurses, engaged with the PHU teams to design the lectures delivered during in loco visits and synchronous training. Finally, the information technology team was responsible for implementing the platform used for teleconsultations and training the PHU teams on the technology used.

### Selection Criteria of PHUs

Initially, a mapping of Brazil’s regions with available vacancies in the *Médicos pelo Brasil* program (a Brazilian federal program that aims to address the significant need for health care professionals in municipalities with high vulnerability, promoting a better distribution of physicians in remote areas of Brazil) was carried out using the reports provided by AgSUS*.* Next, we identified ESF teams with open positions in municipalities within these regions by using the e-Gestor *Atenção Primária à Saúde* (APS) system (a web-based platform that facilitates access to PHC systems and provides strategic information to support the management of programs and services available at the state and municipal levels).

The first selection criterion was that the municipality must have at least one approved ESF team with 50% suspension of resources registered. This ESF team should be without a physician, in accordance with the registration in the *Cadastro Nacional de Estabelecimentos de Saúde* (an operational base for health information systems, responsible for managing the SUS). Furthermore, the municipality should use the e-SUS APS (consisting of a citizen electronic medical record system, which integrates patient care and follow-up data), based on data from the PHC information system. Priority was given to municipalities classified as remote rural areas, which have open physician vacancies.

The following criteria were used for PHUs selection flow for inclusion in the project:

- Availability of a computer with audio and video capabilities, as well as a printer for printing medical prescriptions and issuing certificates
- Stable internet connection with sufficient speed for video calls
- Use of the videoconferencing platform provided by the project, which was installed in advance and for which professionals were trained in its use
- Availability of a private room for teleconsultations
- Support from a local technology team to address infrastructure and system-related issues

### Training Program

#### Synchronous Teletraining

The synchronous teletraining aimed to train the PHU professionals in using the technological tools necessary for teleconsultation operations, clarify the project’s objectives and expected results, and demonstrate the patient’s journey within the PHU in the context of teleconsultation. The training process was structured into (1) technical-operational training, encompassing all aspects of teleconsultation; (2) alignment meetings to assess partial results and identify strategic areas for improvement; and (3) capacity building for professionals involved in the digital PHC service. The teletraining synchronous sessions were scheduled according to the availability of each PHU. Initially, the project team introduced the IConf, the institutional videoconference platform used for teleconsultation. It facilitates communication between patients and physicians, between physicians and ESF team, and between physicians and the management team, following Brazil’s General Data Protection Law (*Lei Geral de Proteção de Dados*). The IConf features included audio or video sharing, screen sharing, presentations with whiteboard and document functions, as well as public and private chat options. Health agents, physicians, dentists, nurses, nursing technicians, PHU managers, and other professionals participated in the synchronous teletraining using the Google Meet platform. In the end, the participants provided anonymous feedback using the Net Promoter Score (NPS) system to assess their satisfaction and likelihood of recommending the service [15].

#### Asynchronous Teletraining

The asynchronous teletraining aimed to provide basic qualifications for “Digital Health at Primary Healthcare” training program. The 20-hour course was available on the HCX e-learning platform of the HCFMUSP and covered the following topics: introduction to digital health in PHUs, regulatory aspects of telemedicine and telehealth, ethical considerations in digital health, legal aspects of Brazilian data protection law, effective patient communication, humanization and media training, humanization of teleconsultations, telepropaedeutics, telepediatrics, teleorthopedics, challenges in teleconsultation in mental health, telerehabilitation, and simulation of teleconsultations. Participants took a test at the end and provided anonymous feedback using the NPS [15] system to assess their satisfaction and likelihood of recommending the course.

#### In Loco Training

In loco training was offered to PHU professionals to increase engagement, facilitate the sharing of experiences, and exchange scientific knowledge. The in loco training included a workshop to discuss common clinical scenarios encountered in PHUs, encompassing clinical issues, work processes, and community-specific aspects. The PHU participants first addressed the case from their respective roles, followed by a theoretical presentation based on their comments and the development of a personalized therapeutic plan for the case. Additionally, the in loco training included a community event aimed at promoting the project, strengthening relationships, and obtaining patient feedback. This event featured a real-time or recorded speech from the PHU physician, emphasizing the importance of medical follow-up. At the end, the participants provided anonymous feedback using the NPS [15] system to assess their satisfaction and probability of recommending the training.

#### Communities of Practice

A community of practice was established for professionals involved in the UBS+Digital project to share their knowledge and experiences related to their telehealth experiences. Through discussing successful cases, participants were able to enhance their understanding of telehealth and identify challenges and opportunities that emerged throughout the project. In order to facilitate this process, an instant group at a messaging app was created for ESF team professionals and local managers to forward their questions. Additionally, synchronous meetings were organized to discuss telehealth experiences from the UBS+Digital project, allowing teams from different PHUs to present their successful cases, meet external speakers, and further engage in collaborative learning. Difficulties and obstacles encountered during the implementation were shared through this group and further discussed at the end of synchronous meetings. Promotion and dissemination of the event occurred through communication channels targeted at professionals within the PHU, emphasizing the importance of this learning opportunity and the exchange of experiences among participants.

#### Technical Visits

The technical visit itinerary included a meeting at the municipal health department aiming to strengthen relationships, update project stakeholders, and promote discussions about the project’s impact on the population and potential action plans. Additionally, the project management team observed the routine of PHU professionals to map out the patient and professional journeys.

#### Teleconsultation Setting

In general, teleconsultations were conducted with patients at the PHU, where the professionals from the ESF team provided on-site assistance. In exceptional cases, home-based teleconsultations were arranged to assist not only patients who were bedridden or mobility impaired but also those living in remote areas or who had not sought care at a PHU. The home visits were conducted using a mobile phone or tablet to enable teleconsultation.

Medical teleconsultation was scheduled either in advance or on the same day, based on the physicians’ availability. If a patient did not attend the appointment, the slot was offered to another patient at the PHU.

The teleconsultations followed the person-centered clinical method and were guided by the Calgary-Cambridge methodology (a structured framework for teaching and assessing medical communication skills, focusing on effective doctor-patient interactions) [16]. When receiving the patient at the web-based platform, the physician confirmed the patient’s full name and birth date. The initial few minutes of interaction were dedicated to establishing clear communication and building a connection.

**Figure 1:**
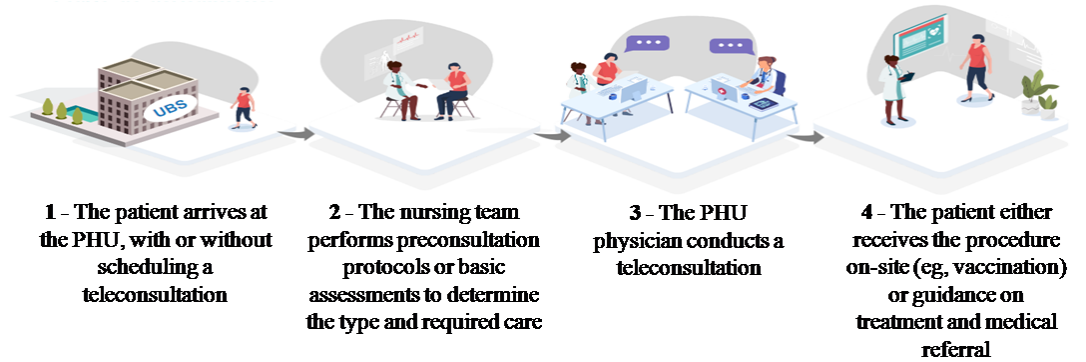
Patient’s journey in the PHU during the telehealth service delivery. PHU: primary health unit.

The projection indicated that teleconsultations would have an average duration of 30 minutes. With 16 hours allocated to this service, the PHU would offer approximately 32 teleconsultations per week. Although a time estimate for consultations was established, physicians had the autonomy to manage the teleconsultation according to the patient’s needs, which could result in a duration shorter or longer than initially projected. Figure 1 illustrates the teleconsultation process.

At the end of each teleconsultation, patients provided anonymous feedback using the NPS system to assess their satisfaction and probability of recommending the service. The physicians completed a web-based form documenting relevant metrics such as consultation duration, number of sessions, unavailability service rate, patient no-shows, and the need for further medical referral.

In addition, teleconsultations and other telehealth care services were categorized, such as clinical case discussion, prescription renewal, reception, teleconsultation at patients’ houses, and counseling.

### KPIs and Project Management

To ensure effective monitoring and management of the UBS+Digital project, a set of predefined KPIs was used to track and evaluate progress. These KPIs were selected based on their relevance to the project’s objectives and were continuously monitored throughout the project’s lifecycle. The KPIs assessed the performance of both training sessions and teleconsultations, with data collected from both patients and health care professionals via structured, web-based surveys. All collected data were securely stored in the REDCap database, maintaining participant anonymity. A dynamic dashboard, developed on the Power BI platform, was used to present real-time updates on key metrics. This allowed the project team to perform continuous monitoring and make informed decisions about the project’s direction. Weekly reviews of the KPIs took place with the project management team to evaluate trends, identify areas needing improvement, and refine strategies. In addition, biweekly meetings with the full project team facilitated deeper discussions and comprehensive data analysis. A resolution rate goal of 50% was set, ensuring the project’s ability to address a substantial proportion of medical cases remotely. Table 1 presents the KPIs that were tracked throughout the project.

**Table 1:** Key performance indicators evaluated during the Unidade Básica de Saúde (UBS)+Digital project.

Key performance indicators analyzed	Description
Number of participants on asynchronous, synchronous, and in loco training	Tracks participation rates in various training formats
NPS^a^ for asynchronous, synchronous, and in loco training	Measures satisfaction with training sessions.
Total number of teleconsultations	Tracks the overall volume of teleconsultations performed
Percentage of service type (teleconsultation, tele-interconsultation, and other services^b^)	Measures distribution of service types provided
Number of patients attended via teleconsultation	Tracks the number of patients served remotely
Percentage of absenteeism	Indicates the rate of missed appointments
Resoluteness rate	Percentage of cases resolved through teleconsultation without the need for in-person referrals
Average duration of teleconsultation	Average length of teleconsultations
NPS for teleconsultations	Measures satisfaction with the teleconsultation experience
Appointment occupancy rate^c^	Percentage of available appointment time used

^a^NPS: Net Promoter Score.

^b^Appointment occupancy rate = occupancy rate (%) = (total time occupied/total available time)×100.

^c^Others services = clinical case discussion, prescription renewal, reception, teleconsultation at patients’ home, and counseling.

### Closure of Operations at PHUs

The closure of operations at the participating PHUs occurred gradually, with a reduction in the number of teleconsultations by the physician to ensure the continuity of care. The transition period lasted an average of 3 weeks, until the total cessation of the service. This process occurred in 3 possible situations: the arrival of a full-time, on-site physician; the end of the project’s execution period; or the lack of participation of essential PHU professionals and managers.

The closure phase started with a meeting between the project team and the municipal health management team to present the transition operation process. During this meeting, data and progress of the specific PHU were reviewed. When applicable, the new physician serving the PHU in person was involved in the process and invited to access the web-based course “Digital Health at Primary Healthcare,” to understand telehealth practices implemented prior to their arrival, thereby highlighting the importance of operational procedures and the quality of patient care.

### Microcosting Analysis

A comprehensive microcosting analysis was conducted to evaluate the economic impact of the UBS+Digital project. The goal was to provide a detailed cost assessment that aligns with the Social Return on Investment (2019) framework, facilitating a clear understanding of the financial sustainability of the project [17]. Data were collected retrospectively, as it was necessary to consolidate productivity data for accurate cost mapping.

The analysis adhered to the Brazilian Economic Evaluation Guidelines of the Ministry of Health [18], following a structured 3-step process:

1. Identification of relevant costs: This involved determining all cost components necessary for the implementation and operation of the project.
2. Measurement of resources used: We quantified the resources consumed by the project at both the implementation and operational stages.
3. Valuation of resources: Each resource was then assigned a monetary value to estimate the total project cost.

The analysis differentiated between implementation costs (one-time costs incurred during project setup) and operational costs (recurring costs associated with ongoing activities). For the implementation phase, cost components included human resources (personnel involved in project setup), infrastructure (physical and technological requirements), e-learning platform (development and deployment costs), and marketing services. The operational phase considered ongoing personnel costs, platform maintenance, and travel expenses for in loco training sessions. To estimate human resource costs, we calculated the number of hours dedicated by each professional, categorizing them into either the implementation phase or the operational phase.

To facilitate decision-making, several key economic metrics were calculated to assess the project’s cost-effectiveness:

- Total cost: The sum of both implementation and operational costs, divided by the total number of teleconsultations performed.
- Actual cost: The real cost per service provided, focusing only on operational expenses.
- Effective cost: The operational costs divided by the number of scheduled teleconsultations, providing insight into cost reductions from improved scheduling.
- Optimized cost: The operational costs divided by an expanded number of teleconsultations, projecting potential efficiency improvements with increased use.
- Break-even time: The time required for the accumulated operational costs to equal the initial investment costs.

This comprehensive microcosting approach allows for the reproducibility of the cost analysis by clearly outlining the steps taken in cost identification, measurement, and valuation. It also provides a transparent framework for estimating the economic impact of similar telehealth initiatives in other regions or countries.

### Statistics Analysis

The data collected anonymously and recorded in the REDCap databases were extracted into Microsoft Excel 2023, where raw values were organized for analysis. Descriptive analysis of the data was performed using Jamovi software (version 2.6.11; Jamovi Project).

The results were expressed both in absolute values and percentages to provide a clear overview of the data distribution. The descriptive analysis involved evaluating frequencies and proportions, allowing for the identification of patterns and general trends within the data set. All results were reviewed to ensure the accuracy and reliability of the conclusions presented in the study.

## Results

### Mapping and Selection of Brazil’s PHUs

All professionals from the project team completed the “Digital Health at Primary Healthcare” course, which explored digital health, including its historical development, definitions, regulatory law, and current challenges, aiming to standardize the knowledge among the professionals regarding the fundamental principles of telehealth services.

**Figure 2:**
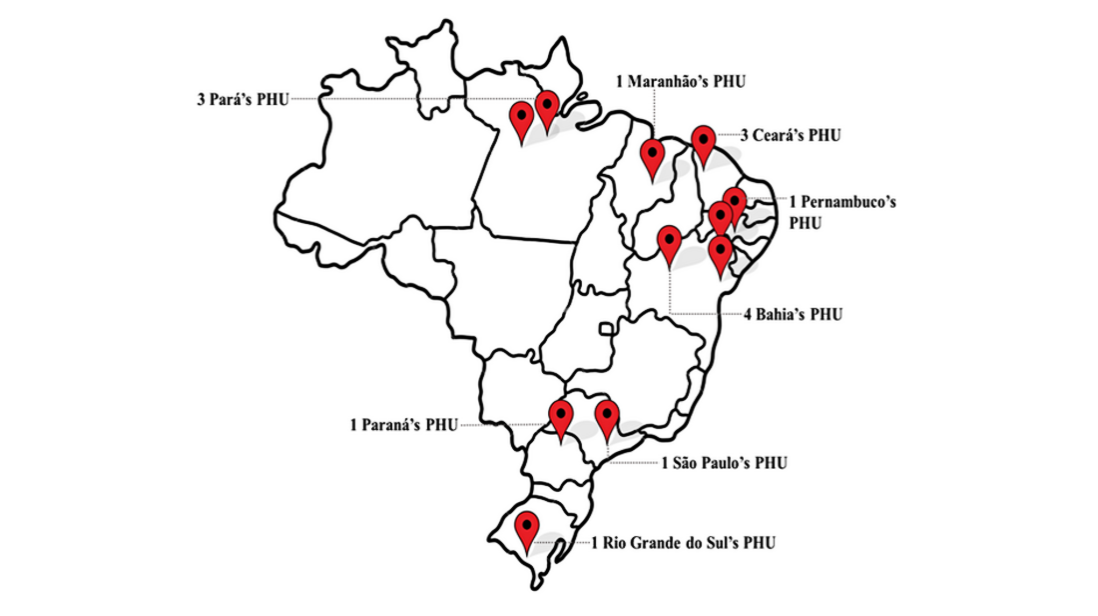
Map of Brazil showing the localization of the 15 selected PHUs. PHC: primary health care.

**Figure 3:**
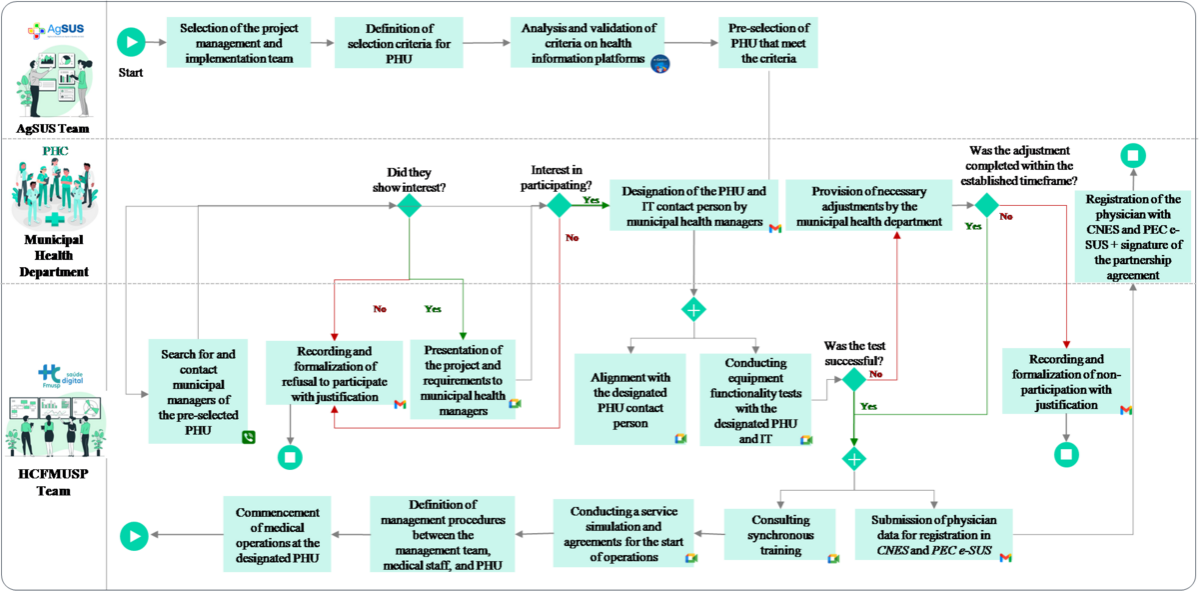
Project implementation workflow. AgSUS: Agência Brasileira de Apoio à Gestão do Sistema Único de Saúde; CNES: Cadastro Nacional de Estabelecimentos de Saúde (National Registry of Health Establishments); HCFMUSP: Hospital das Clínicas da Faculdade de Medicina da Universidade de São Paulo; PEC e-SUS: Prontuário Eletrônico do Cidadão (Electronic Citizen’s Medical Record); PHC: primary health care; PHU: primary health unit.

After searching for ESF teams with open positions in municipalities within the regions mapped for available vacancies in the *Médicos pelo Brasil* program, 322 teams met the first selection criterion: having at least one approved ESF team with a 50% suspension of resources. Of these, 135 municipalities proceeded to a preselection based on additional inclusion criteria outlined for the project. Among these, 39 municipalities expressed interest in taking part in the project, and 25 municipality representatives attended a web-based meeting to discuss the project and its requirements. Ultimately, 11 municipalities agreed to participate in the project. From January to December 2023, the UBS+Digital project served 15 PHUs from 4 regions of Brazil (North, South, Northeast, and Southeast; Figure 2). The median duration of PHU participation in the project was 5 (IQR 1.50-6) months, with the shortest and longest durations being 1 and 7 months, respectively. Figure 3 illustrates the flowchart of the UBS+Digital project implementation at the selected PHUs.

### Monitoring of the PHUs’ Training Program

A total of 153 professionals were synchronously teletrained to use the technological tools necessary for teleconsultation operations. The majority of the professionals were community health agents, community health coordinators, and nursing technicians (Table 2). Approximately 40% (61/153) of professionals responded to the NPS questionnaire, and the NPS score achieved was 100, classified as excellent.

Asynchronous teletraining was offered to a total of 91 professionals, consisting mainly of community health agents’ coordinators, as well as nursing technicians, nurses, managers, and other professionals (buccal health agents, secretaries, administrative and information technicians, community health agents, and dentist; Table 2). Approximately, 19% (17/91) of professionals responded to the NPS questionnaire. The NPS score for this training was 84, classified in the zone of excellence.

Additionally, 9 in loco training sessions were conducted between July and September 2023. Of the 15 PHUs participating in the project, 2 PHUs withdrew before the in loco training, 3 PHUs joined the project without available time to undergo this training and one preferred to complete the training via the web. These training sessions educated 87 professionals, including community health agents, community health coordinators, and nursing technicians (Table 2). The NPS score for this training was 99, classified as excellent.

**Table 2:** Categories of the health care professionals that participated in the synchronous, asynchronous, and in loco training.

Percentage professional	Synchronous (n=153), n (%)	Asynchronous (n=91), n (%)	In loco (n=98), n (%)
Health community agent	76 (50)	48 (53)	48 (44)
Nursing technician	21 (14)	17 (19)	17 (17)
Nurse	16 (10)	10 (11)	9 (9)
Managers	14 (9)	1 (1)	19 (19)
Dentist	3 (2)	2 (2)	2 (2)
Physician	1 (1)	1 (1)	3 (3)
Others^a^	22 (14)	12 (13)	5 (5)
Total	153 (100)	91 (100)	98 (100)

^a^Others: buccal health agents, secretaries, and administrative and information technicians.

The communities of practice strategy was implemented to increase the visibility of the UBS+Digital project and to establish an environment where the professionals involved in the project could share their knowledge and experiences related to telehealth services. To facilitate this, a group was created on an instant messaging app, and web-based meetings were held, in the presence of external speakers. Four meetings were conducted, addressing topics identified by participants as relevant for enhancing teleconsultation practices, such as telemedicine for integrated logistics and telehealth, digital empathy, home care services, hybrid practices linked to health care delivery, and project closure. The first 20 minutes of each session were dedicated to university professors or ESF team professionals who shared successful teleconsultation cases or theoretical topics related to digital health. Following the presentations, a discussion was opened to all participants. It was noted that participants preferred topics related to PHC over more technological subjects. The communities of practice involved PHC managers, health care professionals, the project manager, the teletraining team, the medical project coordinator, physicians, and the project team.

### Monitoring of the Implementation and Impact of Teleconsultation

Initially, the teleconsultation schedule began with a limited offering, and after evaluating the PHU participant adaptation to the teleconsultation system, it was expanded to full capacity (100%). The methodology applied was designed to implement the solution gradually, reducing the necessity for major changes to existing workflows and minimizing the risk of resistance from the local team and patient adherence challenges.

During the telehealth program, a total of 6312 sessions were conducted. Of these, 6140 (97.3%) sessions were teleconsultations; 96 (1.5%) sessions were tele-interconsultations; and 76 (1.2%) sessions were other services such as home visits, renewing medical prescriptions, and medical advice. The teleconsultations served 4279 patients, out of which 1842 (43%) patients returned for follow-up treatment. The teleconsultations had a resolution rate of 85% (5219/6140) and an absenteeism rate of 15% (1083/7223). Most teleconsultations lasted 10 to 20 minutes and were previously scheduled. This service achieved the NPS score of 97, classified as excellent. These results are summarized in Table 3.

**Table 3:** Results of telehealth service offered on the Unidade Básica de Saúde (UBS)+Digital project.

Service offered		Values, n (%)
**Telehealth service (n=6312)**		
	Teleconsultation	6140 (97.3)
	Tele-interconsultation	96 (1.5)
	Others service^a^	76 (1.2)
**Average teleconsultation time (min; n=6140)**		
	0-10	461 (7.5)
	10-20	4054 (66.1)
	>20	1623 (26.4)
**Previous or same-day teleconsultation (n=6140)**		
	Previously scheduled teleconsultation	4009 (65.3)
	Same-day scheduled teleconsultation	2130 (34.7)
Teleconsultation resolution rate		5219 (85)

^a^Other services: clinical cases discussion, prescription renewal, reception, teleconsultation at patients’ house, and counseling.

During the teleconsultation program, 9 technical visits were carried out simultaneously with the in loco training. Initial findings were presented to local stakeholders as health department managers, PHU coordinators, and PHU focal points, providing a project overview and a brief on outcomes from other PHU locations involved.

The operation transition plan was implemented at the end of the teleconsultation operations. Among the 15 participating PHUs, 7 ended their operation due to the arrival of the physician in person, 4 concluded their participation due to the project’s finalization, and 4 lacked engagement. During the teleconsultation transition and arrival of the physician in person, the 7 physicians joining PHUs for in-person activities were able to participate in a teleconsultation and access the web-based course “Digital Health at Primary Healthcare.” The transition process also involved the project physician transferring complex cases, detailing interventions, and setting patient care expectations.

### UBS+Digital Project Microcosting Analysis

To determine the costs of teleconsultations, we considered a total of 6312 telehealth services provided during the study period. The effective cost was calculated based on the total availability of 4752 hours allocated for teleconsultation scheduling, with a maximum operation capacity of 9504 teleconsultations. To estimate the optimized cost, we assumed a 15-minute teleconsultation time, which would allow for an increased capacity of 19,008 teleconsultations within the 4752-hour timeframe. This estimate was based on the observed distribution of teleconsultation durations, where 66.1% (4054/6140) of the teleconsultations lasted between 10 and 20 minutes. The implementation cost was determined using the actual expenses incurred during the setup phase, factoring in an average of 23.6 telehealth services per day. This analysis provides a comprehensive view of cost variations depending on teleconsultation duration and system efficiency, allowing for potential cost optimizations if consultation times are effectively managed. The results of the microcosting evaluation are described in Table 4.

**Table 4:** Project microcosting analysis in different scenarios.

Microcosting indices	Value (US $/teleconsultation)	Time (days)
Total cost	137^a^	—^b^
Real cost	85^a^	—
Effective cost	57^a^	—
Optimized cost	28^a^	—
Time	—	161.5

^a^These results are presented in dollars based on the exchange rate at the time of the project’s implementation.

^b^Not applicable.

## Discussion

### Principal Findings

This study describes the UBS+Digital project, which assessed the implementation and feasibility of a telehealth program in PHUs of the Brazilian Unified Health System, specifically in facilities lacking physicians. The results indicate a high resolution rate of teleconsultations, with a relatively low medical infrastructure cost per consultation. This approach showcased the project’s capability to surmount geographical and economic barriers, particularly in remote areas with limited medical care, physical infrastructure, and internet.

The data showed high patient satisfaction, demonstrated by their return for continued treatment, reinforcing the importance of digital care in building a more resolutive PHC system. One of the main challenges of the UBS+Digital project was establishing the physician-patient relationship that could be enhanced despite geographical distance and the use of technology to access services. Additionally, the project faced the challenge of training and engaging health care professionals, including physicians, nurses, and nursing technicians. This focus on teleconsultation technology and training in specific clinical themes was crucial to the project’s success. Studies have shown that inadequate training for health care professionals can lead to reduced adoption and effectiveness of digital health initiatives [19,20]. The UBS+Digital project mitigated these issues through extensive synchronous, asynchronous, and in loco training, achieving high satisfaction rates among participants.

Moreover, the resolution rate of teleconsultations was high, with minimal referrals, indicating that the majority of health issues were effectively addressed. Accordingly, a study showed that implementing telehealth strategies to support referral management enhanced resolution in PHC and improved care coordination [21]. When combined with professional training and KPI monitoring, teleconsultations have proven effective in other fields, such as tele-ICU, yield better outcomes than initiatives that do not follow this structured approach. These findings suggest adopting well-structured telehealth models—emphasizing proper training and KPI monitoring—in improving consultation resolution and reducing the need for referrals.

A study on the evolution of PHC from 2014 to 2018 documented the increasing implementation of ICTs in health care services [22]. The study described the role of internet access in enhancing care quality and process efficiency. The introduction of these technologies has improved professional practice and health care management. The study also highlighted significant disparities, with a higher concentration of ICT usage in areas with better Human Development Index (HDI) and stronger PHC coverage. However, the North and Northeast regions, which face more challenging economic and social conditions, continue to struggle with inadequate infrastructure and a lack of technological resources. These regional disparities, along with the complexity of implementing telehealth solutions at scale, underscore the need for increased investment in ICT to bridge gaps and ensure equitable health care access. Additionally, the availability of physicians in remote regions remains a challenge, often linked to the HDI by Municipality (HDI-M). The HDI-M measures longevity, education, and income, with values ranging from 0 to 1, where values closer to 1 indicate better development. In this project, the HDI-M of the assessed PHUs ranged from 0.565 to 0.778, indicating a need for increased medical resource allocation [23]. Previous studies have demonstrated that physician availability is directly linked to the HDI-M, reinforcing the necessity of innovative solutions like telehealth to bridge gaps in health care access [24].

The UBS+Digital project showcased the potential of telehealth in addressing health care access challenges in remote areas with limited infrastructure. High resolution rates and positive patient feedback illustrate the effectiveness of telehealth in overcoming geographical and economic barriers, improving access, and ensuring continuity of care. Despite ongoing regional disparities and the need for increased investment in ICT, the project underscores telehealth as a viable solution for underserved areas. However, its scalability is currently limited, and successful implementation requires a balanced approach integrating technological advancements with targeted public policies and strategic investments to reduce regional inequalities and ensure equitable health care coverage. Furthermore, the involvement of human resources and team engagement, including municipal managers and family health teams, were critical to the project’s success.

A comparison between Canada and Brazil highlights shared challenges in health care access due to their vast geographic areas. In both countries, residents of remote areas must travel long distances at significant costs to reach urban centers where medical specialists are concentrated. In Canada, 19% of the rural population faces restricted access to specialized care leading to high socioeconomic burdens and travel-related risks, along with lower availability of medical services [25]. Similar constraints exist in Brazil, particularly in regions with low physician density, further emphasizing the role of telehealth as an essential tool in health care equity [26].

Value-based health care strategy aims to improve patient outcomes by enhancing capacity, comfort, and calm, aligning with the Institute for Health care Improvement’s “triple aim” of improving patient and clinician experiences, population health, and reducing per capita costs [27]. Evidence supports telehealth as a cost-effective model within this framework, as seen in China’s telemedicine program for eye disease screening, which achieved an incremental cost-effectiveness ratio of 34% for traditional methods. In England, telemedicine for acute stroke care led to US $500,000 in cost savings compared with conventional services [27]. Microcosting analyses in other studies align with our findings, showing that optimized teleconsultation workflows reduce costs while maintaining the quality of care [28].

While the initial investment in implementation is significant, evidence indicates that the cost per teleconsultation tends to decrease, reflecting increasing efficiency [29]. Beyond the economic benefits, the project brought significant social value to various stakeholders, including patients, the community, ESF team, the AgSUS and HCFMUSP Digital Health team, and health department managers. For patients and the community, the project raised awareness of the importance of teleconsultations and promoted digital inclusion, enhancing health care access and equity. The project improved the skills of teams at PHUs, AgSUS, and HCFMUSP, particularly in telehealth, applied to PHC, project management, and data analysis. The medical team enhanced their telemedicine skills through courses in digital health for PHC, while also increasing their acceptance of telehealth for medical consultations. For the teams at both AgSUS and HCFMUSP, the project provided valuable insights into the local realities of primary care, enabling more informed decision-making regarding the implementation of telehealth services. Moreover, for municipal health managers, the project offered learning opportunities and insights into training professionals in digital health care. This knowledge significantly strengthens the municipal health system’s ability to effectively adapt to and integrate telehealth solutions.

The implementation of the UBS+Digital project at a PHU in an isolated riverside district exemplifies a significant advancement in health care accessibility with environmental benefits. Previously, a single patient faced a 236-km journey, taking up to 12 hours in one day, to receive in-person medical care in another city. Within just 7 months, the telehealth initiative facilitated 872 teleconsultations at this PHU, achieving an NPS of 96 and resulting in savings of 205,792 km in travel that these individuals would have otherwise needed to undertake by boat and car. The high resolution rate (85%) observed at this PHU indicates that the majority of health issues were effectively addressed, thereby preventing the need for additional travel for follow-up appointments and potentially reducing the carbon footprint associated with such journeys. By minimizing commuting, the project also led to reductions in transportation costs, environmental impact, and risks related to transport accidents. These findings are consistent with global research showing that telehealth initiatives contribute to carbon footprint reduction by decreasing the need for patient travel [30].

This study has certain limitations that should be acknowledged. One key limitation is that we did not conduct a specific analysis of regional differences, which may impact the generalizability of our findings across Brazil’s diverse health care settings. To mitigate this, we established standardized inclusion and exclusion criteria for PHUs to participate in the project. However, despite these criteria, we still encountered significant challenges in infrastructure, internet connectivity, and resource availability, which varied across different units. Additionally, the relatively limited number of PHUs included in the study may not fully capture the wide variability in health care infrastructure, patient demographics, and local health care policies. These factors highlight the complexity of implementing telehealth solutions at scale and underscore the need for future studies to explore strategies to overcome these structural and logistical barriers. Another study limitation is the low NPS response rate, reducing the representativeness of the data. The low response rate may lead to a biased interpretation, as the individuals who chose to respond may not fully represent the broader population of teleconsultation users. Factors such as lack of incentive, time constraints, or unawareness of its importance may contribute to this issue. Consequently, the findings on patient satisfaction and service quality, based on the NPS, should be interpreted with caution, as they might not reflect the experiences of all participants. To mitigate this problem, strategies such as simplifying the questionnaire, sending reminders, and offering incentives have been implemented. Despite these challenges, our findings provide valuable insights into the feasibility and impact of telehealth in primary care settings, contributing to the ongoing development of digital health strategies in Brazil.

### Conclusions

The UBS+Digital project exemplifies how telehealth initiatives can effectively address health care disparities, particularly in underserved regions with limited medical infrastructure. By offering teleconsultations, this project has improved access to health care services, demonstrated a high resolution rate, and achieved notable patient satisfaction that could be considered similar to the model for in-person PHC. The training of health care professionals further ensured the sustainability and quality of these services. These findings highlight the potential for telehealth to mitigate geographic and economic barriers, enhancing health care access in regions where traditional medical resources are scarce. The findings from this study also provide a solid foundation for future research on optimizing telehealth to meet the diverse needs of the Brazilian population. The experience acquired can inform health policies and telehealth implementation strategies in similar global contexts. The microcosting approach used in this study is particularly useful for future evaluations of digital health projects and supports the development of public policies aimed at expanding access to quality health care. Given Brazil’s vast territorial area and significant socioeconomic disparities, telehealth relevance highlights the ongoing need for innovative solutions to improve health care access and quality of services. This highlights the importance of expanding our study in the future to investigate regional disparities, aiming to understand the specific demands and needs of each area.

## Abbreviations

AgSUS: Agência Brasileira de Apoio à Gestão do Sistema Único de Saúde
APS: Atenção Primária à Saúde
ESF: Estratégia Saúde da Família
HCFMUSP: Hospital das Clínicas da Faculdade de Medicina da Universidade de São Paulo
HDI: Human Development Index
HDI-M: Human Development Index by Municipality
ICT: information and communication technology
ICU: intensive care unit
KPI: key performance indicator
NPS: Net Promoter Score
PHC: primary health care
PHU: primary health unit
REDCap: Research Electronic Data Capture
SUS: Sistema Único de Saúde
UBS: Unidade Básica de Saúde

## Data Availability

The datasets generated or analyzed during this study are available from the corresponding author on reasonable request.

## References

[1] (2022). The world’s largest public health system, SUS celebrates 31 years. Gov.BR.

[2] Martins CP (2023). O uso da telemedicina na atenção primária pós-pandemia da COVID-19. The use of telemedicine in primary care after the COVID-19 pandemic. PECIBES.

[3] Mendes EV (2015). A Construção Social da Atenção Primária à Saúde. The Social Construction of Primary Health Care.

[4] Canterle J The number of fully staffed Family Health teams increases in the Federal District. Federal District Health Department.

[5] (2023). Informação e Gestão da Atenção Básica. e-Gestor. Histórico de Cobertura. Information and Management of Primary Care. e-Gestor. Coverage History. Gov.BR.

[6] (2023). Estratégia Saúde da Família, Diário Oficial da União. Portaria de consolidação nº 1, de 2 de junho de 2021/2023. Family Health Strategy, Official Gazette of the Union. Consolidation Ordinance No. 1, of June 2, 2021/2023. Gov.BR.

[7] (2023). Atenção primária à saúde. Primary health care. Pan American Health Organization (PAHO).

[8] Bin KJ, Santana Alves PG, Costa R, Eiras PC, Nader de Araujo L, Pereira AJR, Carvalho C, Malik AM (2023). User experience regarding digital primary health care in santarém, Amazon: evaluation of patient satisfaction and doctor's feedback. JMIR Form Res.

[9] Bashshur RL, Shannon GW, Smith BR, Alverson DC, Antoniotti N, Barsan WG, Bashshur N, Brown EM, Coye MJ, Doarn CR, Ferguson S, Grigsby J, Krupinski EA, Kvedar JC, Linkous J, Merrell RC, Nesbitt T, Poropatich R, Rheuban KS, Sanders JH, Watson AR, Weinstein RS, Yellowlees P (2014). The empirical foundations of telemedicine interventions for chronic disease management. Telemed J E Health.

[10] Aljasim L, Javed NB, Cordoba C, Alyaseen H, Aljasim B, Aljasim M, Cordoba M, Bugis BA, Al-Mohaithef M (2024). Assessing COVID-19 knowledge, attitudes, and practices among hospital employees: identifying sociodemographic determinants for improved public health strategies. Front Public Health.

[11] Halcomb EJ, Ashley C, Dennis S, McInnes S, Morgan M, Zwar N, Williams A (2023). Telehealth use in Australian primary healthcare during COVID-19: a cross-sectional descriptive survey. BMJ Open.

[12] Scudeller PG, Lamas CA, Alvarenga AM, Garcia ML, Amaral TF, de Oliveira MR, de Macedo BR, Testa CB, Baptista FS, Francisco RPV, de Carvalho CRR (2023). Tele-intensive care unit program in Brazil: implementation and expansion. Telemed Rep.

[13] Ministério da Saúde Resolução nº 466, de 12 de dezembro de 2012. Conselho Nacional de Saúde.

[14] Ministério da Saúde Resolução nº 510, de 07 de abril de 2016. Conselho Nacional de Saúde.

[15] Krol MW, de Boer Dolf, Delnoij DM, Rademakers JJDJM (2015). The Net Promoter Score--an asset to patient experience surveys?. Health Expect.

[16] Kurtz S, Silverman J, Benson J, Draper J (2003). Marrying content and process in clinical method teaching: enhancing the Calgary-Cambridge guides. Acad Med.

[17] Teisberg E, Wallace S, O'Hara S (2020). Defining and implementing value-based health care: a strategic framework. Acad Med.

[18] (2014). The Secretariat of Science and Technology and Strategic Inputs of the Ministry of Health and Research in Food and Nutrition.

[19] Scott Kruse C, Karem P, Shifflett K, Vegi L, Ravi K, Brooks M (2018). Evaluating barriers to adopting telemedicine worldwide: a systematic review. J Telemed Telecare.

[20] Gagnon MP, Desmartis M, Labrecque M, Car J, Pagliari C, Pluye P, Frémont P, Gagnon J, Tremblay N, Légaré F (2012). Systematic review of factors influencing the adoption of information and communication technologies by healthcare professionals. J Med Syst.

[21] Gadenz SD, Basso J, de Oliviera PRBP, Sperling S, Zuanazzi MVD, Oliveira GG, da Silva IM, Motta RM, Gehres LG, Pachito DV, de Faria Leao B, de Brito Mallmann, Rodrigues (2021). Telehealth to support referral management in a universal health system: a before-and-after study. BMC Health Serv Res.

[22] Bender JD, Facchini LA, Lapão LMV, Tomasi E, Thumé E (2024). Evolution of the availability of information and communication technologies in primary health care in Brazil, 2012 to 2018. Rev Bras Epidemiol.

[23] (2010). The IBGE Research Skills Development Course.

[24] Carvalho PDD, Barros MVGD, Lima RA, Santos CM, Mélo EN (2011). Health risk behaviors and psychosocial distress indicators in high school students [Article in Portuguese]. Cad Saude Publica.

[25] Agarwal P, Kithulegoda N, Umpierre R, Pawlovich J, Pfeil JN, D'Avila OP, Goncalves M, Harzheim E, Ponka D (2020). Telemedicine in the driver's seat: new role for primary care access in Brazil and Canada: the Besrour papers: a series on the state of family medicine in Canada and Brazil. Can Fam Physician.

[26] Starfield B, Shi L, Macinko J (2005). Contribution of primary care to health systems and health. Milbank Q.

[27] Teisberg E, Wallace S, O'Hara S (2020). Defining and implementing value-based health care: a strategic framework. Acad Med.

[28] Henderson C, Knapp M, Fernández JL, Beecham J, Hirani SP, Cartwright M, Rixon L, Beynon M, Rogers A, Bower P, Doll H, Fitzpatrick R, Steventon A, Bardsley M, Hendy J, Newman SP (2013). Cost effectiveness of telehealth for patients with long term conditions (Whole Systems Demonstrator telehealth questionnaire study): nested economic evaluation in a pragmatic, cluster randomised controlled trial. BMJ.

[29] Huguet M, Sarazin M, Perrier L, Augusto V (2022). How we can reap the full benefit of teleconsultations: economic evaluation combined with a performance evaluation through a discrete-event simulation. J Med Internet Res.

[30] Rutherford JS, Sherwin ED, Ravikumar AP, Heath GA, Englander J, Cooley D, Lyon D, Omara M, Langfitt Q, Brandt AR (2021). Closing the methane gap in US oil and natural gas production emissions inventories. Nat Commun.

[31] ChatGPT. OpenAI.

